# Intraspecific Variations in Functional and Molecular Traits of Near-Endemic *Onopordum alexandrinum* Boiss. in Natural and Anthropogenic Habitats along the Western Mediterranean Coast of Egypt: Implications for Conservation

**DOI:** 10.3390/plants9081041

**Published:** 2020-08-16

**Authors:** Aya Yahia, Ibrahim Mashaly, Magdy El-Bana, Rehab Rizk, Ghada El-Sherbeny

**Affiliations:** 1 Department of Botany, Faculty of Science, Mansoura University, Mansoura 35516, Egypt; aya.latif@mans.edu.eg (A.Y.); iamashaly1950@mans.edu.eg (I.M.); rehabrizk@mans.edu.eg (R.R.); ghada204@mans.edu.eg (G.E.-S.); 2 Department of Botany, Faculty of Sciences, Port Said University, Port Said 42654, Egypt

**Keywords:** conservation, functional traits, gene flow, ISSR, RAPD, *Onopordum alexandrinum*

## Abstract

*Onopordum alexandrinum* is a near-endemic to Egypt and neighboring countries. Its habitats are designated as priority sites for conservation in the south and east Mediterranean regions. We hypothesize that variation in morphological, reproductive, and molecular traits could provide a survival strategy that allows the species to occupy landscapes including anthropogenic habitats (barley and abandoned fields) and natural habitats (sand dunes and desert plateau) with different soil resources along the western Mediterranean coast of Egypt. The results indicated that plant functional traits associated with high rates of resource acquisition and growth (e.g., high values of vegetative height and specific leaf area, and low values of leaf dry matter content) occurred in populations located in abandoned fields with high soil resources. The genetic diversity analyses indicated similarity in genetic diversity of the present populations of *O. alexandrinum* in barley and abandoned fields with those of sand dunes. However, the genetic structures of these populations were different from those of natural desert plateau, which suggests reduced rates of gene flow. In this framework, it is essential to monitor and reduce the anthropogenic activity which will not only support the conservation of genetic diversity within populations but will also help ensure the resilience of *O. alexandrinum* in the face of environmental and climatic changes.

## 1. Introduction

Understanding the patterns and processes associated with changes in functional and genetic traits is one of the key goals in the preservation and conservation of rare and threatened species [1]. In this context, the concepts of plant functional traits and genetic diversity have received great attention in conservation ecology in response to human-induced changes such as rapid habitat loss and climate change [2,3,4]. These traits are defined as morpho-physio-phenological and molecular characteristics that impact the fitness of individual species via their effects on plant adaptation, survival, and growth [5,6]. Moreover, functional and molecular traits can indicate how a species relates and responds to its environment, which offers a powerful approach to conservation and ecological questions of rare and endangered plant species [7,8,9].

Understanding environmental forces (i.e., soil, topographic properties, and light environment) responsible for changes in functional traits and genetic diversity is important in species conservation because it reflects how a population survives under fluctuated conditions (e.g., changing resources and soil nutrients) [5,10,11]. Such forces can influence a population’s performance directly by changing resource availability or indirectly by changing the growth rate and reproductive effort of plants, which are determined by an interaction between resources and conditions and the functional and genetic traits of plants [12,13,14]. Variations in these traits are the reflection of evolutionary and environmental drivers that operate at different spatial and temporal scales and are difficult to differentiate within and among plant populations, especially in endangered and threatened species because of the low number of individuals [5,13,15,16]. Understating this differentiation is important because of its potential to help us better address fundamental questions, such as how rare and threatened species become extinct, how they disperse and spread, and how their communities are assembled.

Investigation of intraspecific variation in functional and genetic traits may further improve the accuracy and resolution of studies of conservation and community ecology [17,18]. This intraspecific trait variation can have important implications for monitoring and conserving threatened and rare species [19,20,21] and maintaining ecosystem functions [22,23]. Phenotypic plasticity (i.e., the production of multiple phenotypes from a single genotype) can be partly responsible for intraspecific variation [24]. Such plasticity through morphological and/or physiological adaptation enables plants to adjust their performance in response to changes in environmental conditions throughout their lifespan [15,24]. Moreover, differences in the genotype and heritable differences in gene expression and function contribute to intraspecific species variation [17,25]. Since rare and threatened species are known for their limited and narrow distribution, it is likely that finer-scale trait variation plays an important role in controlling species establishment and persistence [26]. Classic techniques investigating the morphological, physiological, and biochemical traits that confer tolerance to environmental stress have recently been integrated with other analytical tools, such as genetic diversity analyses. Molecular markers have been proved to be a valuable tool in the characterization and evaluation of the genetic diversity within and between natural populations in a species [27]. For example, it has been shown that some markers can reveal different classes of polymorphism [28,29]. Other molecular markers can detect genetic variation, identify ecotypes and species with a relatively low level of intraspecific polymorphism, and can indicate the overall tolerance of a species to environmental fluctuations [30,31].

*Onopordum alexandrinum* Boiss. (Asteraceae) is a plant near-endemic to Egypt and neighboring countries [32,33,34], where its habitats are designated as priority sites for conservation along the south and east Mediterranean regions [35]. In Egypt, the size of *O. alexandrinum* populations has been dramatically reduced and become highly fragmented by natural and anthropogenic factors such as climate change and habitat destruction. According to our survey, the populations exist in natural habitats (sand dunes and desert plateaus) and in anthropogenic habitats (barley crops and abandoned fields). Despite its threatened status due to reductions of population size and limited geographic distribution, no research has focused on the population structure and genetic diversity of this species. Therefore, resolving the intraspecific variation in both functional and molecular traits of *O. alexandrinum* is a priority for plant conservationists in the south and east Mediterranean regions. As the two approaches provide different but complementary information, an understanding of both aspects of trait variability and pattern is desirable for the development of appropriate conservation strategies. Accordingly, we hypothesize that variation in morphological, reproductive, and molecular traits could provide a survival strategy that allows the species to occupy landscapes including anthropogenic habitats (barley and abandoned fields) and natural habitats (sand dunes and desert plateau) with different soil resources along the western Mediterranean coast of Egypt. In this context, the following questions were addressed: (1) Are there intraspecific variations in morphological and reproductive traits among populations of *O. alexandrinum* in anthropogenic and natural habitats? (2) How large is the genetic diversity within populations and genetic differentiation among these populations? (3) Do local soil resources affect these variations? (4) Is it possible to draw general conclusions for long-term monitoring and the establishment of a conservation strategy for *O. alexandrinum*?

## 2. Results

### 2.1. Soil Properties

**Figure 1:**
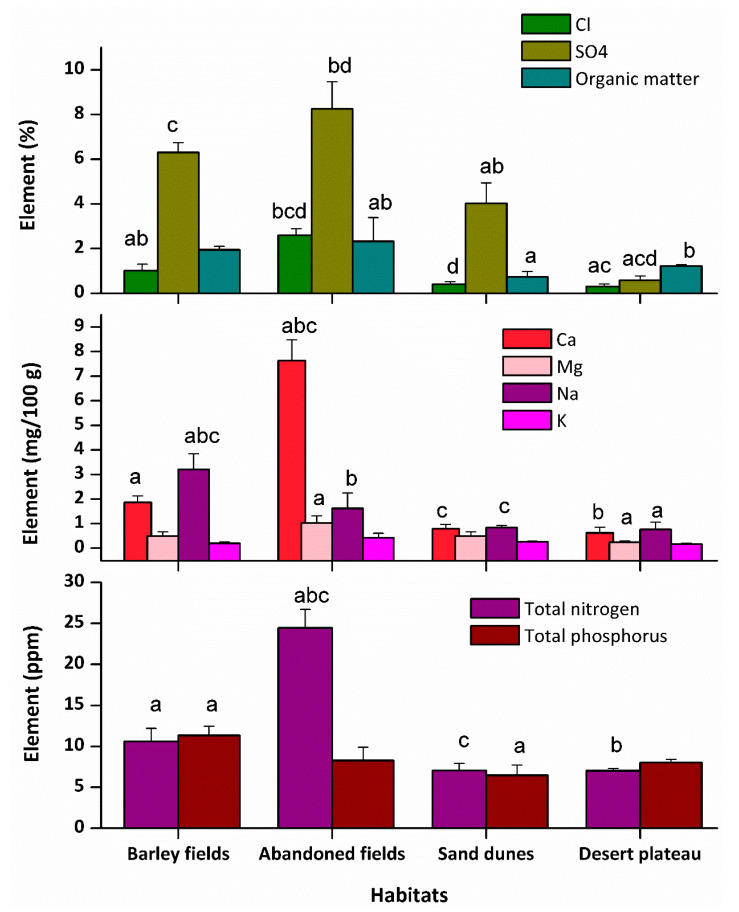
Comparisons of soil variables at anthropogenic and natural habitats of *Onopordum alexandrinum*. Bars with similar letters within each soil variable are significantly different according to Tukey’s studentized range test at the 0.05 probability level.

Most of the measured soil variables were significantly different in the four habitats (Figure 1). The soil of the abandoned fields was clearly distinguished from other habitats by greater percentages of Cl^−^, SO_4_^−−^, and organic matter (2.6%, 8.25%, and 2.33%, respectively), as well as contents of total nitrogen (24.46 ppm) as well as Ca^++^ and Mg^++^ (7.63 and 1.03 mg/100 g, respectively). The soil of the desert plateau was at the opposite end of resource availability, with the lowest percentages of Cl^−^ and SO_4_^−−^ (0.3% and 0.57%, respectively) as well as Mg^++^ content (0.25 mg/100 g). It was obvious that the barley fields had the highest values of Na^+^ (3.21 mg/100 g) and total phosphorous (11.33 ppm). The desert plateau had the opposite trend, with the lowest content of Ca^++^ and Na^+^ (0.62 and 0.77 mg/100 g, respectively).

### 2.2. Functional Traits

**Figure 2:**
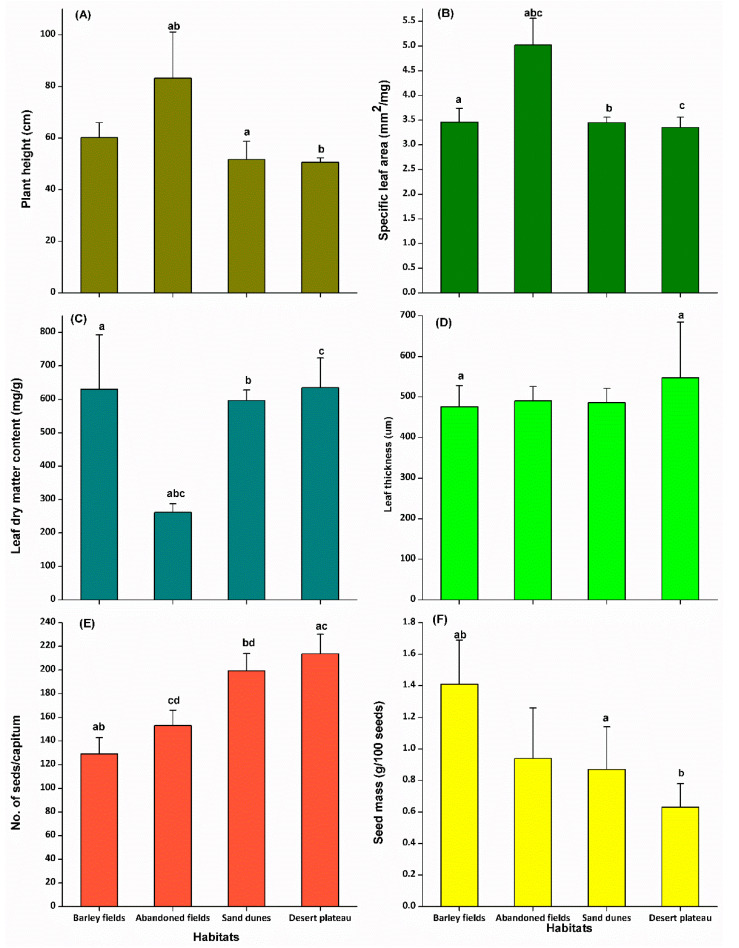
Comparisons of functional traits of *Onopordum alexandrinum* in anthropogenic and natural habitats. Bars with similar letters are significantly different according to Tukey’s studentized range test at the 0.05 probability level. (**A**): Plant height; (**B**): Specific leaf area; (**C**): Leaf dry matter content; (**D**): Leaf thickness; (**E**): Number of seeds; (**F**): Seed mass.

The populations of *O. alexandrinum* showed significant differences for all the measured traits (Figure 2). Individuals from abandoned fields had the highest vegetative height (83.13 cm) and the largest specific leaf area (5.02 mm^2^/mg) but the lowest leaf dry matter content (261.4 mg/g). Individuals from desert plateau had the shortest height (50.67 cm), highest leaf dry matter content (634.05 mg/g), thickest leaves (547.27 μm), highest seed production (213.33 seeds/capitulum), the lowest specific leaf area (3.35 mm^2^/mg), and the lowest seed mass (0.63 g/100 seeds). Individuals from barley fields had the lowest number of seeds (129 seeds/capitulum) but the heaviest seed mass (1.41 g/100 seeds).

### 2.3. Trait–Soil Correlation

The correlation results indicated that the significant drivers of soil-trait relationships were Cl^−^, SO_4_^−−^, Ca^++^, Mg^++^, total nitrogen, and total phosphorus (Table 1). All of these variables were significantly associated with at least one trait. Specific leaf area correlated positively with soil SO_4_^−−^ (*r* = 0.47), Ca^++^ (*r* = 0.55), Mg^++^ (*r* = 0.61), total nitrogen (*r* = 0.65), and total phosphorus (*r* = 0.49). Leaf dry matter content was correlated with both soil Ca^++^ (*r* = 0.48) and total P (*r* = 0.51). Among the reproductive traits, seed number was clearly related to all measured variables except SO_4_^−−^, K^+^, and total P.

### 2.4. Molecular Analysis

The six random amplified polymorphic DNA (RAPD) primers and six inter simple sequence repeat (ISSR) primers produced 32 and 45 unambiguous and reproducible bands, respectively (Table 2). In the case of RAPD, the molecular sizes of bands ranged from 200 bp to 1100 for the primer PO-A7. The minimum number of bands per primer was four (for each of OP-A9, OP-A18, and OP-C4), while the maximum number of bands per primer was seven (for each of OP-A7 and OP-B7). In the case of ISSR, the sizes of bands ranged from 255 bp (ISSR 49A) to 1065 bp (ISSR 44A). The minimum number of bands per primer was five (ISSR HB-09), while the maximum number of bands per primer was eleven (ISSR 44A).

**Figure 3:**
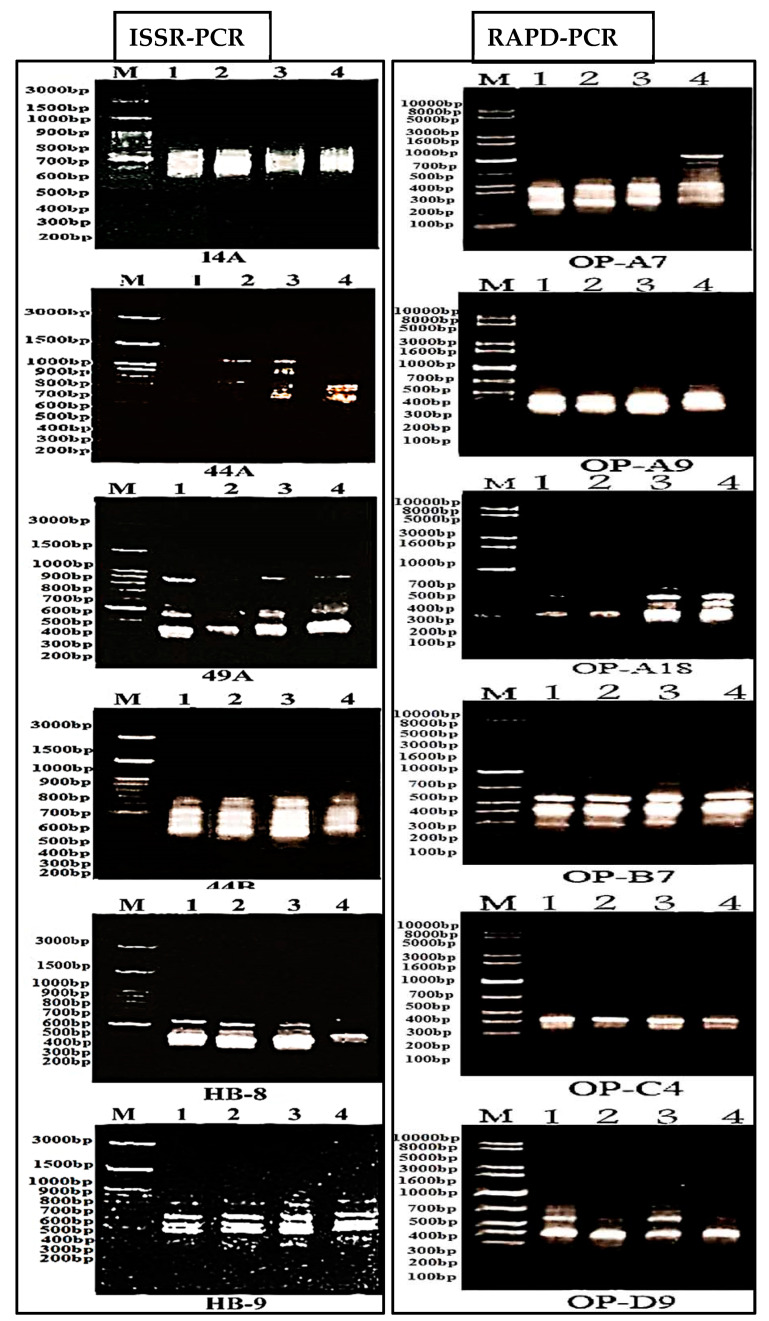
Random amplified polymorphic DNA (RAPD) and inter simple sequence repeat (ISSR) profiles of *Onopordum alexandrinum* genotypes generated with different six primers. Lanes 1–5 represented the sample habitats. M = Molecular size marker. 1 = Oa1 (sand dunes), 2 = Oa2 (abandoned fields), 3 = Oa3 (barley fields), 4 = Oa4 (desert plateau).

The total percentage of polymorphism of ISSR markers (53.33%) was higher than that of RAPD markers (31.25%) (Table 3). In addition, the percentages of polymorphic loci were higher for ISSR markers, with values of 22.22% in sand dunes (Oa1), 17.78% in abandoned fields (Oa2), 26.67% in barley field (Oa3), and 28.89% in desert plateau (Oa4); however, their comparable values of RAPD were 12.5%, 0%, 21.88%, and 12.5%, respectively. The extent of polymorphism observed among the four *O. alexandrinum* habitats as revealed by various RAPD and ISSR primers is shown in Figure 3.

**Figure 4:**
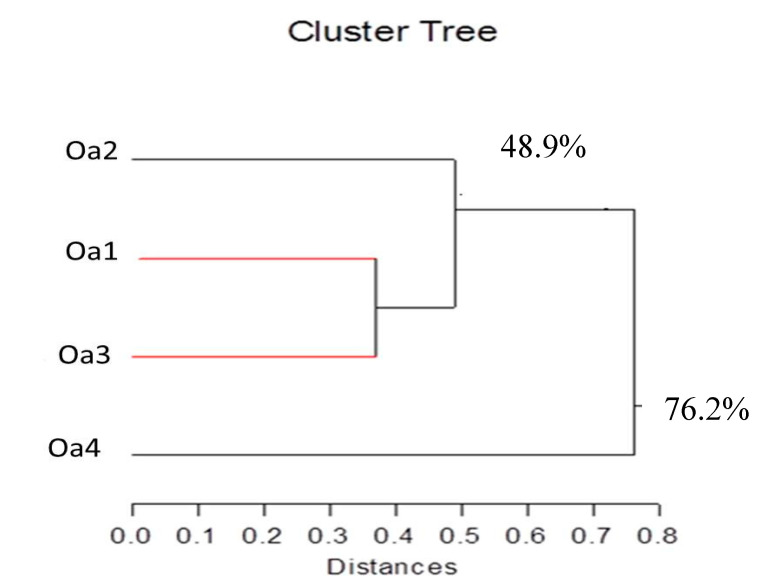
Cluster dendrogram representing genetic relationship among the four accessions of *Onopordum alexandrinum* collected from different habitats. 1 = Oa1 (sand dunes), 2 = Oa2 (abandoned fields), 3 = Oa3 (barley fields), 4 = Oa4 (desert plateau).

The RAPD and ISSR data were combined for cluster analysis. The obtained cluster dendrogram is shown in Figure 4. A dendrogram constructed on the polymorphism data separated the habitats into two distinct clusters at 76.2% variation, with the first cluster including the desert plateau (Oa4) accession separated from the second cluster comprised of habitats of sand dunes (Oa1), abandoned fields (Oa2), and barley fields (Oa3). The second cluster is further divided into two sub-clusters at 48.9% variation, where the accession sub-cluster of abandoned fields (Oa2) was detached from the habitats sub-cluster of sand dunes (Oa1) and barley fields (Oa3). The similarity correlation coefficients ranged from 0.20 (Oa2 and Oa4) to 0.63 (Oa1 and Oa3) (Table 4).

## 3. Discussion

Our results indicated that *O. alexandrinum* is characterized by intraspecific phenotypic variation in morphological traits (plant height, specific leaf area, and leaf dry matter content), which is consistent with previous studies (e.g., assessment of 769 herbaceous species of the British flora [36]). The populations of *O. alexandrinum* in abandoned fields had significantly higher plant height and specific leaf area compared to other habitats. These findings might be related to the significantly higher availability of nutrients (Cl^−^, SO_4_^−−^, Ca^++^, Mg^++^) along with higher contents of organic matter and total nitrogen. Specific leaf area was found to be positively correlated with the availability of soil SO_4_^−−^, Ca^++^, Mg^++^, total N, and total P, as also demonstrated experimentally by Al Haj Khaled et al. [37]. Wright et al. [38] also documented that the availability of high nitrogen concentrations is integral to the production of the proteins involved in the photosynthetic machinery that resulted in increasing plant height and specific leaf area. Such a pattern can be interpreted in the frame of Coley’s resource availability hypothesis, which relates larger leaf turnover, faster growth, and generally higher tolerance to environmental stress on more fertile patches [39,40,41].

Wright et al. and Reich [38,42] argued that a functional trade-off as described by the leaf economics spectrum is related to differences between species growth rate strategies. The main trade-off is between a resource acquisition strategy and a resource conservation strategy. The ability to photosynthesize more and grow rapidly represents the first strategy, for example, through higher specific leaf area and lower leaf dry matter content. On the other hand, the ability to cope with low resource environments, for instance, through lower specific leaf area and higher leaf dry matter content, represents the second strategy. In our study, different populations of *O. alexandrinum* demonstrated the two strategies of phenotypic plasticity in the different habitats, with the highest specific leaf area and the lowest leaf dry matter content in the abandoned fields, representing the first strategy, while the opposite was in the case the natural population of the desert plateaus, which representing the second strategy. In addition, the leaves of desert plateau populations had high leaf dry matter content and were thicker than those of abandoned fields. Cornelissen et al. and Kleyer et al. [40,43] demonstrated that high values of leaf dry matter content correspond to a low turnover rate and thicker leaves that are better adapted to physical stress, such as thin and low soil nutrients in desert plateau. Both characteristics represent adaptations that provide a big advantage for conserving nutrients in low resource environments [44]. Gross et al. [45] reported that leaf dry matter content tends to be a better predictor for plant responses to physical stress than specific leaf area. However, specific leaf area tends to be high in nutrient-rich habitats like abandoned fields, thereby potentially resulting in high relative growth rate, photosynthetic efficiency, and fast turnover of plant parts, which allow phenotypic adaptation to the spatial patchiness of resource availability [40,43,46].

Seed production and seed mass normally display opposite trends in different habitats [47,48,49]. Therefore, a plant population with high seed production tends to have low seed mass due to the known trade-off between these components [50]. The populations of *O. alexandrinum* in desert plateaus had the highest seed production and the lowest seed mass, while those in barley fields had large seeds but low seed production. These results confirm intraspecific phenotypic variation in the seed traits of *O. alexandrinum*. The low seed production of *O. alexandrinum* in disturbed barley fields could be related to setting fewer seeds when facing increased environmental constrains or competition from crowded barley and other species, while larger seed mass will allow higher survival, establishment, and growth rates for the seedling [47]. Seedlings from larger seeds survive better in disturbed, competitive habitats than those from smaller seeds [49,51]. Kitajima and Myers [52] also confirmed that larger seeded species may be more competitive and tolerate stress better than smaller-seeded species, though smaller-seeded species produce a greater number of seeds [50,51]. Therefore, the seed mass is typically a very strong predictor of growth and survival rates [49].

The molecular diversity analysis for the accessions of *O. alexandrinum* indicated that the range and average polymorphic banding pattern of ISSR were higher than those of RAPD. This indicates that ISSR markers were superior to the RAPD marker in their capacity of providing more informative bands, as found in other studies [53,54,55,56]. This can be related to ISSR technologies having lower sensitivity of PCR amplification [54], more reproducibility [57], greater effectiveness at uncovering polymorphism [58], higher stability [55], and a higher mutation rate [58]. However, other studies reported that RAPD markers were more polymorphic than ISSR in different plant species [59,60,61,62]. This contrast between the two markers could be connected to differences in genome composition and the proportion of coding and non-coding within the genome of a species.

In our study, the values of the polymorphic markers of ISSR and RAPD in *O. alexandrinum* are comparable with those reported for other rare and endemic Asteraceae [63,64,65]; however, these values are lower than those documented for other widespread and invasive *Onopordum* species [66,67] and Asteraceae [68,69]. Generally, species with wide geographic distribution tend to acquire higher genetic diversity than those with limited geographic ranges, such as rare and endemic species [22,70]. The genetic diversity within and among plant populations are controlled by several factors, including life-history traits, breeding type, reproductive mode, geographic distribution, and environmental stress, which are determinants to adaptation and evolution as well as the effective and efficient conservation of rare species [71,72,73]. Consequently, conservation efforts should be focused on natural populations that maintain the overall genetic variation of the target species. The cluster analysis and correlation coefficients between the genetic accessions revealed that the populations of *O. alexandrinum* in the study area were largely affected by anthropogenic disturbance and the geographical distribution of the accessions. The populations from anthropogenic habitats (barley and abandoned fields) with the adjacent sand dunes were clearly clustered together and separated from the natural population from desert plateau. This suggests that the populations of these anthropogenic fields could have originated from the surrounding sand dunes [74]. The presence of these nearby populations without topographical barriers may facilitate the pollination and dispersal of seeds and consequently result in more gene flow with lower genetic and geographic distances among neighboring populations [75,76]. On the other hand, the separation of the farthest and most distinct cluster of desert plateau from the cluster of other habitats indicated lower gene flow and exchange through pollination or seed dispersal among populations.

### Implications for Conservation 

Anthropogenic activity has led to a dramatic reduction in the natural habitats of *O. alexandrinum* along the western Mediterranean coast of Egypt. Therefore, the current study focused on the understanding of its ecology and genetic diversity, which influence a population’s ability for survival within different habitats, in order to direct its conservation programs. The debate about whether ecological factors contribute more than genetic factors in driving species to extinction has been recently quantitatively assessed for different endangered species [77]. Many threatened plant taxa are affected adversely by genetic factors, as indicated by research in anthropogenic and fragmented habitats [78,79]. These approaches involve the integration of population genetics, functional traits, and landscape ecology as part of a multidisciplinary framework that will provide knowledge-based tools for conserving the evolutionary potential of species and for managing ongoing anthropogenic modified landscapes [80]. Our results indicate that some functional traits of *O. alexandrinum* responded to the changes in soil resources modified by anthropogenic activity. Therefore, plant functional traits associated with high rates of resource acquisition and growth, such as high values of vegetative height and specific leaf area, and low values of leaf dry matter content and seed mass, were found in the populations of abandoned fields with high soil resources. Conversely, populations with low values of specific leaf area and vegetative height and high values of leaf dry matter content and seed mass were recorded in desert plateaus with low soil nutrient levels. Similarly, plant genetic traits are affected by the creation of rich habitat patches of barley and abandoned fields within regions that formerly supported only natural populations of *O. alexandrinum*. Therefore, natural populations of sand dunes exist and remain fragmented among the populations of barley and abandoned fields, as indicated by similarity in genetic diversity and the lack of genetic differentiation. In other words, the present populations of *O. alexandrinum* in barley and abandoned fields were likely to be derived from previously large populations of sand dunes. This suggests that increasing gene flow to the anthropogenic fields may lead to increasing mate availability and a broadening of the genetic base among these populations, which may increase the ability of *O. alexandrinum* to survive local selection pressures. On the other hand, the results indicated that the gene structures of these populations are different from those of populations from natural desert plateau, which suggests reducing rates of gene flow, which may lead to the deterioration of diversity in local populations of *O. alexandrinum*. The genetic distance between the sand dune and desert plateau populations could imply that intraspecific hybridization of both genotypes in restoration sites could result in increases in the number, size, and fitness of populations, and consequently aid in the conservation of *O. alexandrinum*. In this framework, the preservation of the genetic structure and maintenance of the unique genetic traits of *O. alexandrinum* populations may depend on maintaining a balance between isolation from and connectivity with neighboring populations [13]. In this context, it is essential to monitor and reduce the anthropogenic activities that induce changes to the resources of natural habitats and to the rates of gene flow among populations.

## 4. Materials and Methods

### 4.1. Study Area and Habitat Selection

The study was carried out along the western sector of Mediterranean coastal land of Egypt that extends for about 550 km between Alexandria westward to Sallum at the Libyan border and with an average width of 20 km in a north–south direction [81]. The landscape of this region was divided into a northern coastal plain and a southern tableland, with a chain of calcareous dunes and rocky ridges [82]. The climate is classified as arid Mediterranean, with dry summers and mild winters [83]. The rainy season extends from October to March, with the mean annual rainfall ranging from 191 mm to 417 mm at Alexandria and Matrouh, respectively, while the mean annual temperature is 20.6 °C and 19.5 °C, respectively [84].

We selected two anthropogenic habitats (barley and abandoned fields) and two natural habitats (sand dunes and desert plateau), which represent the main distribution of *O. alexandrinum* along the western Mediterranean coast of Egypt. The barley and abandoned fields are the most fertile habitats, which are located on the sandy plains and depressions in the study area [74]. The barley fields are either irrigated by underground water or rainfed farming systems. The abandoned fields are lands that were previously cultivated for barley, figs, and olives but are now abandoned and occupied by natural vegetation. In each habitat, a total of three sampling plots (5 m × 5 m) were randomly selected to represent the micro-variations of *O. alexandrinum*. The field survey and measurements were carried out during March 2018.

### 4.2. Soil Analysis

Three composite soil samples were collected randomly from three different locations (0 to 50 cm depth) within each plot and subsequently pooled for each plot. Soil samples were brought to the laboratory, air dried at room temperature, and sieved through a 2-mm sieve. The estimation of organic carbon was carried out using air dried soil samples. Chlorides, sulfates, total N, total P, and extractable cations (Na^+^, K^+^, Ca^++^ and Mg^++^) were determined according to Allen [85].

### 4.3. Functional Traits

The trait sampling followed standard procedures [40,43]. For each selected plot in each habitat, we randomly selected ten robust well-grown adult individuals without symptoms of infection or herbivory damage. The plant vegetative height from the ground was measured in the field. Then, we collected three leaves from each of the ten measured individuals, which were brought back to the laboratory in an ice-cool box, while three capitula were collected and placed in paper bags. Leaf area (LA = one-sided projected area) was measured on five fresh samples from the longest full-grown leaves of five individuals using portable laser leaf area meter CI-202 scanning planimeter (CID Inc., Camas, WA, USA). After measurements of the area and fresh weight, the leaves were oven-dried at 70 °C for 48 h. Based on these measurements, we calculated the following morphological traits: specific leaf area (SLA = ratio of leaf area to leaf dry mass) and leaf dry matter content (LDMC = ratio of leaf dry mass to saturated fresh mass). Leaf thickness was estimated from the ratio (SLA × LDMC) − 1, as described by Vile et al. [86]. Capitula were air-dried and manually cleaned. Next, the seeds were counted and weighed using a balance with an accuracy of 0.1 mg to obtain seed mass.

### 4.4. Molecular Traits

#### 4.4.1. Sample Collection and DNA Extraction

Young leaf samples were collected from 36 isolated populations of *O. alexandrinum*, which were chosen to represent nine populations for each of the four sampled habitats. Leaf material was randomly collected from 10–20 individuals from each population. The collected leaf material was stored with silica gel in zip-lock plastic bags until use. Total genomic DNA was extracted from each accession following the standard method, as described by Dellaporta et al. [87]. The leaves were ground in liquid nitrogen, homogenized, and preserved at −80 °C until further use. DNA concentration was determined by both spectrophotometry and gel electrophoresis.

#### 4.4.2. Random Amplified Polymorphic DNA (RAPD) Amplification

RAPD analysis was carried out using six random 10-mer arbitrary primers synthesized by Operon biotechnologies, Inc., Germany, as shown in Table 5. The DNA amplifications were performed following the procedures of Williams et al. [88,89]. The DNA amplifications were performed in an automated thermal cycle (model Techno 512) programmed for one cycle at 94 °C for 4 min, followed by 45 cycles: denaturation at 94 °C for 1 min, annealing at 37 °C for 1 min, and extension at 72 °C for 2 min, and the reaction was finally held at 72 °C for 10 min. The PCR products were separated on agarose gel and photographed with a digital camera using a transilluminator, then scanned and analyzed with Bio-Rad Video Gel documentation 2000 [90].

#### 4.4.3. Inter Simple Sequence Repeat (ISSR) Amplification

The ISSR primers and their sequences are presented in Table 5. The DNA amplifications were performed in an automated thermal cycle (model Techni 3000G) programmed with initial denaturation of one cycle at 94 °C for 4 min, followed by 45 cycles: denaturation at 94 °C for 1 min, extension at 57 °C for 1 min, at 72 °C for 2 min, and finally held at 72 °C for 10 min. The PCR products were separated on agarose gel and photographed with a digital camera using a transilluminator, then scanned and analyzed with Bio-Rad Video Gel documentation 2000 [90].

### 4.5. Data Analysis

One-way analysis of variance (ANOVA) was used to test the significance of variations in the soil variables and the measured morphological and reproductive traits in the different studied habitats. Simple linear correlation coefficient (*r*) was calculated in order to evaluate the type of relationship between the measured functional traits (morphological and reproductive) and estimated soil variables.

The RAPD and ISSR bands were scored visually for the presence (1) or absence (0) of bands of various molecular weight sizes. The DNA fingerprint patterns obtained from RAPD and ISSR analyses were converted into binary data matrices containing arrays of 0s and 1s. Only polymorphic and reproducible bands were considered for the analysis. Data were analyzed using cluster analysis to generate a dendrogram illustrating the relationships among the four populations of different habitats based on biochemical and molecular data. The percentage of polymorphic bands was determined according to the following equation:

$$\%\;\mathrm{Polymorphism}=\frac{\sum \;\mathrm{bands}\;\mathrm{for}\;\mathrm{each}\;\mathrm{sample}\;-\;\sum \;\mathrm{bands}\;\mathrm{for}\;\mathrm{all}\;\mathrm{species}}{\sum \;\mathrm{bands}\;\mathrm{for}\;\mathrm{all}\;\mathrm{species}}\times 100$$

All data analyses were performed using SYSTAT version 7.0 [91].

## 5. Conclusions

Populations of natural habitats such as sand dunes and desert plateaus should be considered a high priority for conservation in order to expand population size and genetic diversity. Such an action will not only be an effective manner of supporting the conservation of genetic diversity within populations but will also help to ensure resilience in the face of environmental and climatic changes.

## Figures and Tables

**Table 1:** Simple linear correlation coefficient (*r*) between the soil variables and functional traits of *Onopordum alexandrinum*. SLA: Specific Leaf Area; LDMC: Leaf Dry Matter Content. Bold values indicate significant at *p* < 0.05.

Plant Traits	Soil Variables								
	Cl^−^	SO_4_^−−^	Ca^++^	Mg^++^	Na^+^	K^+^	OM	Total N	Total P
Plant height	−0.25	−0.01	−0.09	−0.02	−0.12	0.14	0.01	−0.27	−0.30
Leaf thickness	−0.21	**0.53**	−0.40	−0.10	−0.12	0.25	0.20	−0.25	−0.38
SLA	−0.37	**0.47**	**0.55**	**0.61**	−0.36	0.14	−0.01	**0.65**	**0.49**
LDMC	0.34	−0.44	**0.48**	0.31	0.11	−0.22	−0.20	0.40	**0.51**
Seed number	**−0.58**	0.17	**−0.63**	**−0.69**	**−0.85**	−0.19	**−0.58**	**−0.64**	−0.42
Seed mass	0.06	−0.13	−0.08	−0.34	0.15	0.12	0.17	0.05	0.09

**Table 2:** Profiles of RAPD and ISSR primers used for the study genetic diversity of different habitats of *Onopordum alexandrinum*. 1 = Oa1 (sand dunes), 2 = Oa2 (abandoned fields), 3 = Oa3 (barley fields), 4 = Oa4 (desert plateau).

**RAPD**				
**Genus**	**Onopordum Alexandrinum**			
Code	Oa1	Oa2	Oa3	Oa4
Bp	OP-A7			
1100	0	0	0	1
1000	0	0	0	1
450	1	1	1	1
400	1	1	1	1
280	1	1	1	1
260	1	1	1	1
200	1	1	1	1
Amplified bands	5	5	5	7
	OP-A9			
630	0	0	0	1
480	1	1	1	1
300	1	1	1	1
230	1	1	1	1
Amplified bands	3	3	3	4
	OP-A18			
700	0	0	1	0
500	1	1	1	1
400	1	0	1	1
300	1	1	1	1
Amplified bands	3	2	4	3
		OP-B7		
1000	0	0	1	0
700	1	0	1	0
580	1	1	1	1
450	1	1	1	1
400	1	1	1	1
390	1	1	1	1
290	1	1	1	1
Amplified bands	6	5	7	5
	OP-C4			
580	1	0	1	0
500	1	1	1	1
400	1	1	1	1
360	1	1	1	1
Amplified bands	4	3	4	3
	OP-D9			
1000	0	0	1	0
930	1	0	1	0
800	1	1	1	1
700	1	1	1	1
570	1	1	1	1
400	1	1	1	1
Amplified bands	5	4	6	4
**ISSR**				
**Genus**	**Onopordum Alexandrinum**			
Code	Oa1	Oa2	Oa3	Oa4
Bp	14 A			
660	1	1	1	1
585	1	1	1	1
530	1	1	1	1
440	1	1	1	1
400	0	1	0	0
390	1	0	0	0
375	0	0	0	0
Amplified bands	5	5	4	4
	44A			
1065	0	1	1	0
1040	0	0	0	1
865	0	0	1	0
835	0	0	0	1
690	1	1	1	1
600	1	0	1	1
535	0	0	1	1
530	1	0	0	0
475	1	0	1	1
460	0	1	0	0
390	0	0	0	1
Amplified bands	4	3	6	7
	49A			
885	1	1	1	1
615	0	0	0	1
540	0	0	0	1
450	1	0	1	1
400	1	1	1	1
285	1	1	1	1
255	1	0	1	1
Amplified bands	5	3	5	7
	44B			
970	0	0	0	1
620	1	1	1	1
560	1	1	1	1
475	1	1	1	0
440	1	1	1	1
385	1	1	1	1
325	1	1	1	1
295	1	1	1	1
Amplified bands	7	7	7	7
	HB-08			
545	0	1	1	0
500	0	0	0	1
470	1	1	1	0
440	0	0	0	1
390	1	1	1	0
350	1	1	1	1
315	1	1	1	1
285	1	1	1	0
Amplified bands	5	6	6	4
	HB-09			
730	1	1	1	1
570	1	1	1	1
480	1	1	1	1
430	1	1	1	1
335	1	1	1	1
Amplified bands	5	5	5	5

**Table 3:** The range of band products, and the number of common and polymorphic bands produced by different primers for *Onopordum alexandrinum* populations generated by six primers. 1 = Oa1 (sand dunes), 2 = Oa2 (abandoned fields), 3 = Oa3 (barley fields), 4 = Oa4 (desert plateau).

Primers		Range of Band Products (bp)	Total Bands	Polymorphic Bands				
				Total	Oa1	Oa2	Oa3	Oa4
**RAPD**	**OP-A7**	200–1100	7	2	0	0	0	2
	**OP-A9**	230–630	4	1	0	0	0	1
	**OP-A18**	300–700	4	2	1	0	2	1
	**OP-B7**	290–1000	7	2	1	0	2	0
	**OP-C4**	360–580	4	1	1	0	1	0
	**OP-D9**	400–1000	6	2	1	0	2	0
	Total bands		32	10	4	0	7	4
	Percentage (%)			31.25	12.5	0	21.88	12.5
**ISSR**	**14 A**	390–660	6	2	1	1	0	0
	**44A**	390–1065	11	10	3	2	5	6
	**49A**	255–885	7	4	2	0	2	4
	**44B**	295–970	8	2	1	1	1	1
	**HB08**	285–545	8	6	3	4	4	2
	**HB09**	335–730	5	0	0	0	0	0
	Total bands		45	24	10	8	12	13
	Percentage (%)			53.33	22.22	17.78	26.67	28.89

**Table 4:** Similarity coefficients of the four habitats of *Onopordum alexandrinum* collected from four different habitats. 1 = Oa1 (sand dunes), 2 = Oa2 (abandoned fields), 3 = Oa3 (barley fields), 4 = Oa4 (desert plateau). Bold values indicate significant at *p* < 0.05.

Accessions	Oa1	Oa2	Oa3	Oa4
**Oa1**	1.000			
**Oa2**	**0.513**	1.000		
**Oa3**	**0.630**	**0.509**	1.000	
**Oa4**	0.300	0.200	0.213	1.000

**Table 5:** Nucleotide sequences of RAPD and ISSR primers used for the study of the genetic diversity of *Onopordum alexandrinum* habitats. The primers were synthesized by Operon Biotechnologies Inc. (Cologne, Germany).

RAPD		ISSR	
Codes	Nucleotide Sequence 5’to 3’	Codes	Nucleotide Sequence 5’to 3’
OP-A7	5’ GAA AGG GGT G 3’	14A	CTC TCT CTC TCT CTC TTG
OP-A9	5´ GGG TAA CGC C 3’	44A	CAC ACA CAC ACA AG
OP-A18	5´AGG TGA CCG T 3’	49A	GTAGATTATGTTCCTTCTCC
OP-B7	5´ GAA AGG GGT G 3’	44B	CTC TCT CTC TCT CTC TGC
OP-C4	5´ CCG CAT CTA C 3’	HB8	GAG AGA GAG AGA GG
OP-D9	5´ GGT GAC GCA G 3’	HB9	GTG TGT GTG TGT GG
